# Induction of Antibodies and T Cell Responses by a Recombinant Influenza Virus Carrying an HIV-1 TatΔ_51–59_ Protein in Mice

**DOI:** 10.1155/2014/904038

**Published:** 2014-05-14

**Authors:** B. Garulli, G. Di Mario, M. G. Stillitano, D. Compagnoni, F. Titti, A. Cafaro, B. Ensoli, Y. Kawaoka, M. R. Castrucci

**Affiliations:** ^1^Department of Infectious, Parasitic and Immune-Mediated Diseases, Istituto Superiore di Sanità, 00161 Rome, Italy; ^2^Department of Biology and Biotechnology “Charles Darwin”, Sapienza University of Rome, 00185 Rome, Italy; ^3^National AIDS Center, Istituto Superiore di Sanità, 00161 Rome, Italy; ^4^Department of Pathobiological Sciences, School of Veterinary Medicine, University of Wisconsin-Madison, Madison, WI 53711, USA; ^5^Division of Virology, Department of Microbiology and Immunology and International Research Center for Infectious Diseases, Institute of Medical Science, University of Tokyo, Tokyo 108-8639, Japan; ^6^Infection-Induced Host Responses Project, Exploratory Research for Advanced Technology, Saitama 332-0012, Japan

## Abstract

Recombinant influenza viruses hold promise as vectors for vaccines to prevent transmission of mucosal pathogens. In this study, we generated a recombinant WSN/TatΔ_51–59_ virus in which Tat protein lacking residues 51 to 59 of the basic domain was inserted into the N-terminus of the hemagglutinin (HA) of A/WSN/33 virus. The TatΔ_51–59_ insertion into the viral HA caused a 2-log reduction in viral titers in cell culture, compared with the parental A/WSN/33 virus, and severely affected virus replication *in vivo*. Nevertheless, Tat-specific antibodies and T cell responses were elicited upon a single intranasal immunization of BALB/c mice with WSN/TatΔ_51–59_ virus. Moreover, Tat-specific immune responses were also detected following vaccine administration via the vaginal route. These data provide further evidence that moderately large HIV antigens can be delivered by chimeric HA constructs and elicit specific immune responses, thus increasing the options for the potential use of recombinant influenza viruses, and their derivatives, for prophylactic and therapeutic vaccines.

## 1. Introduction

The continuous spread of HIV-1 calls for efforts to develop vaccines that are protective and capable of eliciting both cellular and humoral immunity at systemic and mucosal sites. Several studies suggest that the abatement of HIV replication at mucosal surfaces is likely to be essential for the success of a prophylactic vaccine and that the local induction of high levels of high-avidity mucosal CD8+ T cells with antigen protective specificities may be able to prevent, at early times of infection, the steady and massive spread of virus from the mucosa into the systemic circulation [1–4]. Antienvelope responses can play an important protective role, and antibodies to early HIV regulatory gene products, including Tat, Rev, and Nef, are expected to impact HIV acute infection. In particular, the Tat protein is produced very early after HIV-1 infection and is necessary for viral gene expression and disease progression [5]. This protein is well conserved among the circulating HIV-1 clades, and cross-sectional and longitudinal studies of natural infection suggest that the presence of an anti-Tat immune response correlates with asymptomatic infections and a slower progression to disease [6–8]. It has been also shown that Tat-specific cytotoxic T lymphocytes (CTL) play a role in controlling the early virus infection [9]. For these reasons, a native Tat protein-based vaccine has been developed and tested in human clinical trials [10–13].

Influenza A viruses are strong inducers of humoral and cellular immune responses, and live recombinant influenza viruses have proven to be suitable vectors to present foreign antigens to the immune system in mice. Effective immune responses against foreign epitopes recognized in the context of class I and class II MHC molecules have been largely described upon infection by recombinant influenza viruses given via intranasal (i.n.) route [14–16]. Moreover, previous studies have shown that mucosal and systemic immune responses are also induced upon vaginal (i.vag.) immunization of progesterone-treated female mice with recombinant influenza viruses [17].

Here, we generated a recombinant influenza virus (WSN/TatΔ_51–59_) by inserting a Tat protein that lacked residues 51 to 59 of its basic domain into the N-terminus of the HA of A/WSN/33 (H1N1) (WSN) virus. This coding region of* tat *gene was removed to prevent recombination events at the HA cleavage site and also to reduce the possibility of interactions between these basic residues and host cell factors during transit through the secretory pathway. We then characterized the humoral and cellular immune responses elicited upon a single immunization via either the i.n. or i.vag. route in mice. In this way, we could explore the strategy to accommodate moderately large HIV antigens on the viral surface as HA fusion proteins and examine their immunogenic properties, thus increasing the options for the potential use of recombinant influenza viruses as a platform for innovative vaccines. In particular, we provide evidence for a new Tat-based vaccine that may prove useful in establishing effective mucosal immunization protocols for HIV-1.

## 2. Materials and Methods

### 2.1. Construction of the pPolI-WSN HA/TatΔ_51–59_ Plasmid and Generation of Recombinant WSN/TatΔ_51–59_ Virus

The nucleotide sequence encoding 86 amino acids of Tat protein (HIV-1 IIIB, BH-10 clone), flanked by ClaI and PstI restriction sites, was inserted into the cloning cassette previously generated at the 3′ end of the signal peptide coding sequence of the pPolI-WSN HA plasmid, yielding the pPolI-WSN HA/Tat plasmid [16]. To delete the region coding for residues 51 to 59 (KRRQRRRPP), the pPolI-WSN HA/Tat plasmid was amplified by inverse PCR and back-to-back Tat-specific primers, yielding the pPolI-WSN HA/TatΔ_51–59_ plasmid [18]. RT-PCR conditions, including primer sequences, are available on request. Plasmids constructs were sequenced to ensure that unwanted mutations were not introduced by PCR and the cloning procedures.

The recombinant influenza virus WSN/TatΔ_51–59_ bearing the TatΔ_51–59_ insertion in its HA was generated by use of plasmid-driven reverse genetics, as described by Neumann et al. [19]. At 48 h after transfection, the virus was harvested, plaque-purified in MDCK cells, and then inoculated into MDCK cells to make virus stocks. To determine the stability of the chimeric HA/TatΔ_51–59_ gene construct, WSN/TatΔ_51–59_ virus was passaged ten times on MDCK cells, and the presence of the insert was confirmed by direct sequencing of RT-PCR products using primers for the HA gene [5′-GGCAAAACTACTGGTCCTGT-3′ (forward), 5′-TACTGAGCTCAATTGCTCCC-3′ (reverse)].

### 2.2. Western Blot Analysis

Western blot analysis of proteins obtained from virus concentrated through a 20% sucrose cushion was performed by electrophoretic transfer of the proteins from the 12% polyacrylamide gel to a PVDF-membrane (Immobilon P, Millipore) at 250 mA for 90 min. After being blotted, the membrane was incubated for 2 h with a cocktail of monoclonal antibodies to the HA1 of WSN virus or with a specific rabbit anti-Tat hyperimmune serum (diluted 1 : 200 in TBS) (Diatheva, Fano, Italy) for 2 h at room temperature. The membrane was then washed three times and incubated for 1 h at room temperature with HRP-goat anti-mouse or goat anti-rabbit IgG diluted 1 : 1.000, and immunocomplexes were detected by using the ECL-substrate system (Amersham Pharmacia Biotech).

### 2.3. Immunofluorescence

MDCK cells were infected with WSN/TatΔ_51–59_ or WSN virus at a multiplicity of infection of 0.1. Twenty-four hours after infection (p.i.), the cells were washed, fixed in PBS containing 4% paraformaldehyde, and incubated for 40 min with mouse monoclonal antibodies specific to the HA of WSN virus or a specific rabbit anti-Tat hyperimmune serum (Diatheva). After being extensively washed with PBS, cells were incubated for 30 min with fluorescein isothiocyanate conjugated to goat anti-mouse IgG or goat anti-rabbit IgG (Sigma), respectively.

### 2.4. Immunization of Mice

All animal work wasperformed in compliance with institutional guidelines and approved protocols. Female BALB/c mice were lightly anesthetized with Avertin (2,2,2-tribromoethanol) before being i.n. infected with 45 *μ*L of PBS containing 10^5^ plaque-forming units (PFU) of WSN/TatΔ_51–59_ virus or 10^3^ PFU of WSN virus. For i.vag infection, groups of mice were subcutaneously injected with 3 mg of progesterone (Depo-Provera; Pharmacia & Upjohn) because of the influence of the estrous cycle on viral infectivity. Five days later, the mice were synchronous in the progestinic phase and could support vaginal replication upon i.vag. injection with 10^5^ PFU of WSN/TatΔ_51–59_ virus or 10^3^ PFU of WSN virus in a 10 *μ*L volume [17].

### 2.5. Viral Replication in Murine Respiratory and Genital Tracts

To assess viral replication in mouse respiratory tracts, a total of three groups of BALB/c mice (*n* = 4/group) were immunized under anesthesia and sacrificed on days 1, 3, and 5 after i.n. inoculation of 10^5^ PFU of WSN/TatΔ_51–59_ virus. Control groups of mice were i.n. infected with 10^3^ PFU of WSN virus and sacrificed on the same days. Lungs were aseptically removed and tissue-derived extracts were prepared by grinding the tissue samples in a homogenizer to a 10% (wt/vol) suspension in PBS. The suspensions were then centrifuged at 3,000 ×g for 5 min, and the supernatants were assayed for infectious virus particles by 48 h incubation of serial dilutions of samples with permissive MDCK cells, followed by standard hemagglutination with chicken red blood cells. The endpoint titers were estimated in triplicate measurements by interpolation and expressed as total TCID_50_/organ.

To assess viral replication in the vaginal mucosa of mice immunized via the i.vag. route, vaginal lavages were collected daily for eight days by rinsing the vaginal cavity twice with 50 *μ*L of sterile PBS and were stored at −80°C until testing. Virus titers were determined with MDCK cells, as described above.

### 2.6. IFN-*γ*-Specific ELISPOT Assay

BALB/c mice were immunized via the i.vag. or i.n. route and spleens and lymph nodes draining the respiratory tracts (mediastinal lymph nodes, MLN) and the vaginal tracts (iliac lymph nodes, ILN), respectively, were collected on day 7 p.i. and used to isolate single-cell lymphocyte populations, as previously described [20]. Antigen-specific IFN-*γ*-producing cells were enumerated by using a commercially available murine IFN-*γ* ELISPOT kit (BD, Pharmingen). Briefly, fresh lymphocytes from distinct pools of spleens and lymph nodes were added to 96-well ELISPOT plates precoated with the IFN-*γ*-specific capture antibody and incubated at 37°C for 24 h untreated or in the presence of VCF (VCFITKALGISYGRK) Tat peptide (residues 36 to 50) or influenza nucleoprotein NP147 peptide (residues 147 to 155: TYQRTRALV) (H-2K^d^) [21–23]. Colored spots representing IFN-*γ*-releasing cells are reported as the number of spot-forming cells (SFC) per 10^6^ cells.

### 2.7. Tat-Specific T Cell Proliferation

Splenocytes (4 × 10^5^/200 *μ*L) were cultured in 96-well plates in the presence or absence of affinity-purified and biologically active Tat protein (0.1, 1, 5, or 10 *μ*g/mL) for 5 days at 37°C. [^3^H] thymidine (Amersham Biosciences, UK) (0.5 *μ*Ci/well) was added 12–15 h before harvesting. The stimulation index (S.I.) was calculated by dividing the mean counts/min of five wells of antigen-stimulated cells by the mean counts/min of the same cells grown in the absence of antigen.

### 2.8. Detection of Tat-Specific Antibodies

Serum samples were examined for antigen-specific IgG antibodies by use of an enzyme-linked immunosorbent assay (ELISA). Blood samples were collected by retroorbital plexus puncture before immunization, at 21 days after single i.n. infection with WSN or WSN/TatΔ_51–59_ virus, and at 14 days after boost. Then, 96-well microtiter plates (Nunc-Immuno Plate MaxiSorp; Nunc Life Technologies) were coated overnight at 4°C with 200 *μ*L of Tat protein (0.5 *μ*g/mL in 0.05 M carbonate buffer pH 9.6). The plates were washed and saturated with blocking buffer (PBS containing 0.05% Tween 20 and 1% BSA) for 1.5 h at 37°C. After five washes, 100 *μ*L of test serum from individual mice was serially diluted in blocking buffer, added to duplicate wells, and incubated for 1.5 h at 37°C. The wells were then washed again and incubated for 1.5 h at 37°C with 100 *μ*L of HRP-goat anti-mouse IgG serum (Sigma). After a final round of washes, bound antibodies were detected by the addition of 100 *μ*L of TMB (Vector), and absorbance was read at a wavelength of 450 nm.

## 3. Results

### 3.1. Generation and Growth Properties of a Recombinant Influenza Virus Expressing TatΔ_51–59_ Protein

**Figure 1:**
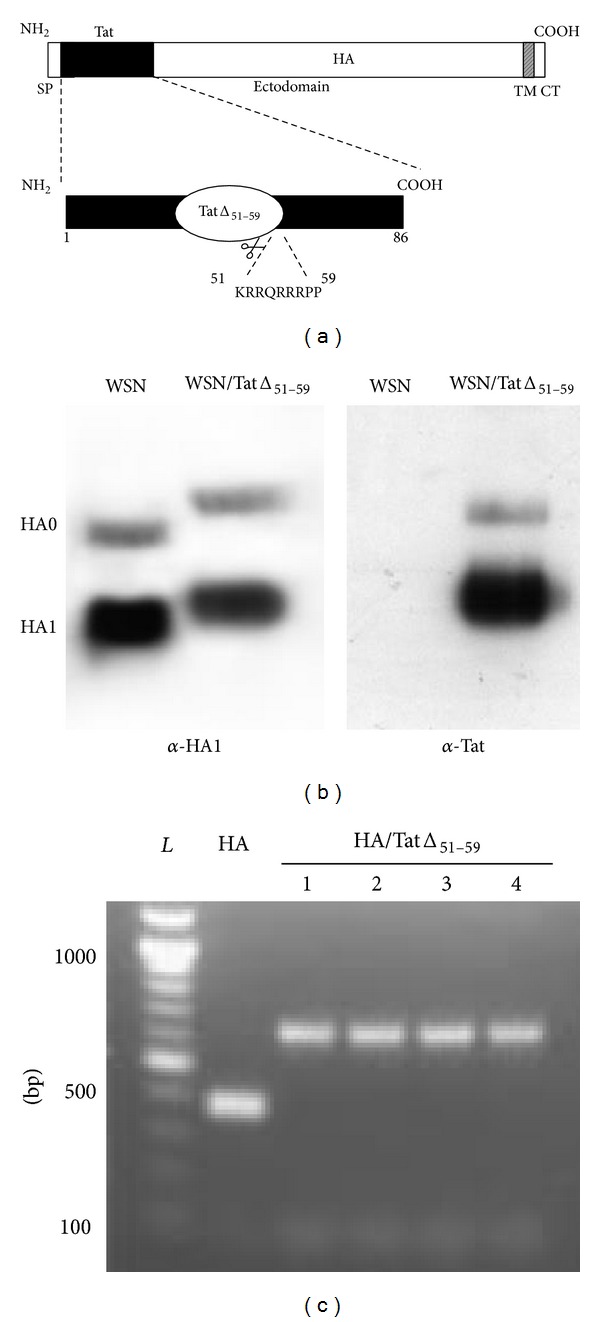
Construction and expression of WSN/TatΔ_51–59_ virus. The coding sequence of the Tat protein of HIV IIIB (BH-10 clone), that lacked residues 51 to 59 of the basic region, was inserted into the cloning cassette of the plasmid pPolI-WSN HA, after the HA signal peptide (SP). CT, TM = cytoplasmic, transmembrane domains of HA, respectively (a). Viral protein lysates were separated on a 12% polyacrylamide gel and analyzed in Western blots by using mouse monoclonal antibodies specific to the HA1 of WSN virus or a specific rabbit anti-Tat hyperimmune serum (b). Genetic stability of WSN/TatΔ_51–59_ virus was determined in four different plaques by RT-PCR after six passages on MDCK cells. *L* = 100 bp DNA ladder (c).

The chimeric HA/TatΔ_51–59_ construct that lacked residues 51 to 59 of the basic region of Tat was generated, and the recombinant influenza virus, designated WSN/TatΔ_51–59_, was rescued from cDNA by using reverse genetics [19] (Figure 1(a)). The WSN/TatΔ_51–59_ virus was plaque-purified in MDCK and then inoculated into MDCK cells to make a virus stock. The nucleotide sequence of the viral RNA confirmed the in-frame insertion of the nucleotides encoding the Tat protein lacking residues 51 to 59, fused at the N-terminal end of HA.

To determine whether the Tat sequence was incorporated into the virions, WSN and WSN/TatΔ_51–59_ viruses were analyzed by using 12% SDS-PAGE, followed by Western Blotting with a mixture of anti-HA1 monoclonal antibodies, and a polyclonal serum specific for the Tat protein. The migration patterns of the HA0 precursor and HA1 of WSN/TatΔ_51–59_ virus were consistent with the molecular size expected for the chimeric HA/TatΔ_51–59_ protein (Figure 1(b)). Moreover, the reactivity, or lack thereof, of the WSN/TatΔ_51–59_ and WSN viruses, respectively, with the anti-Tat-specific serum confirmed the specificity of the insertion into the chimeric HA.

**Figure 2:**
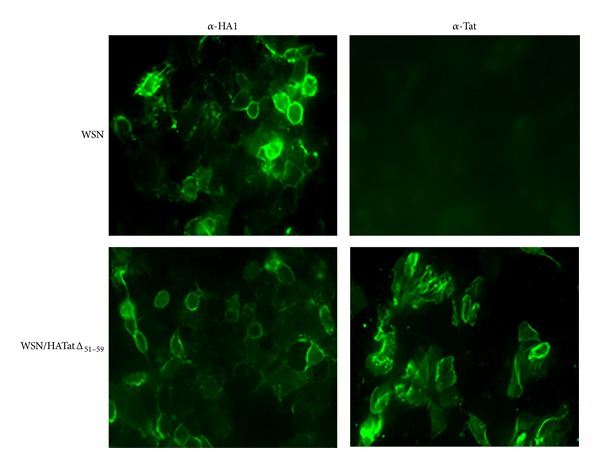
Immunofluorescence analysis. MDCK cells were infected with WSN or WSN/TatΔ_51–59_ virus. At 24 h p.i., the cells were stained with monoclonal antibodies specific for WSN H1 HA protein or a specific rabbit anti-Tat hyperimmune serum. They were then incubated with fluorescein isothiocyanate conjugated to goat anti-mouse IgG or goat anti-rabbit IgG, respectively.

**Figure 3:**
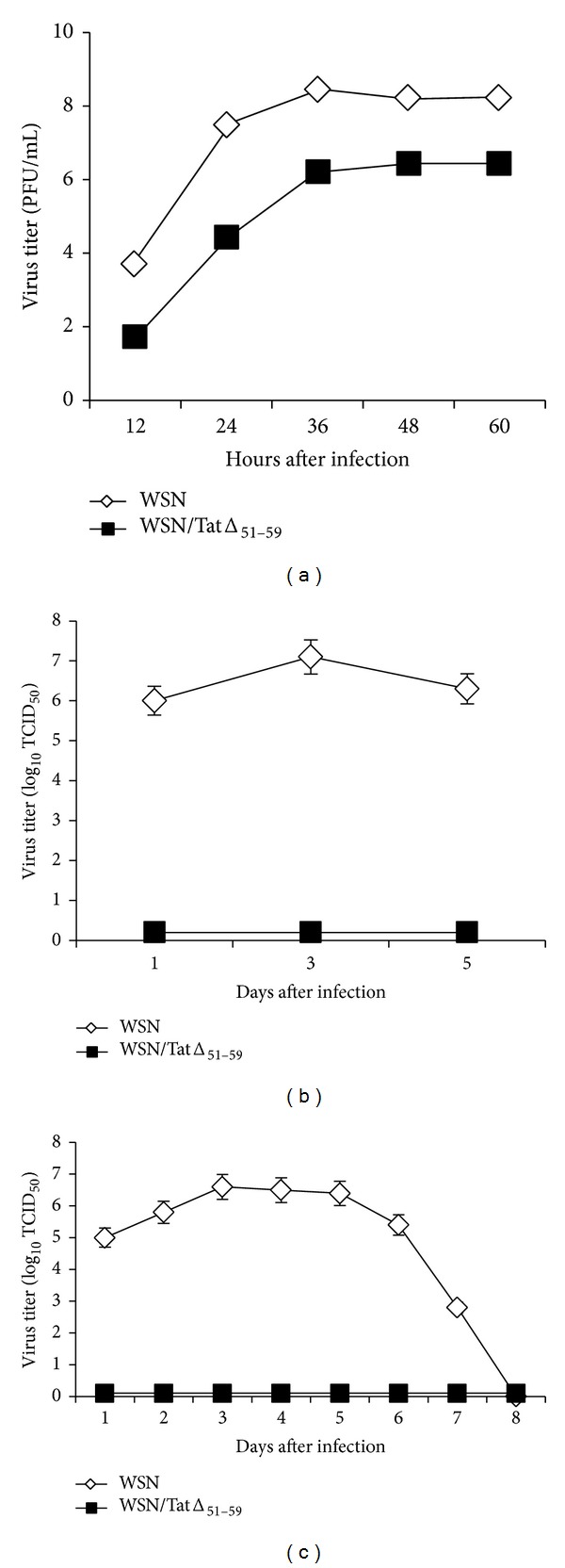
*In vitro* and* in vivo* replication of WSN/TatΔ_51–59_ virus. Growth properties of WSN and WSN/TatΔ_51–59_ viruses in MDCK cells. The viruses were used to inoculate MDCK cells at a multiplicity of infection of 0.01, and at the indicated times after infection virus titers in the supernatants were determined (a). Three groups of BALB/c mice (*n* = 4/group) were i.n. infected with 10^3^ PFU of WSN virus and three other groups of mice (*n* = 4/group) with 10^5^ PFU of WSN/TatΔ_51–59_ virus. Animals from each group were euthanized on days 1, 3, and 5 p.i., and lungs were collected from individual mice for viral titration on MDCK cells (b). Progesterone-treated mice were i.vag. infected with the above viruses, and viral titers in vaginal washes from individual mice were determined at the indicated time points by endpoint titration in MDCK cells (c). Bars represent means ± standard deviation (SD) for four mice per group.

The growth kinetics of the recombinant WSN/TatΔ_51–59_ virus in MDCK cells were reduced compared with those of the wild-type WSN virus, reaching viral titers of 10^6.5^ PFU/mL and 10^8.2^ PFU/mL, respectively (Figure 3(a)). The Tat insertion into the HA gene, revealed by PCR from different plaques after ten passages of the WSN/TatΔ_51–59_ virus in tissue culture, indicates the stable retention and expression of the foreign antigen in the recombinant influenza virus (Figure 1(c)). The presence of the Tat antigen in infected MDCK cells was also analyzed by immunofluorescence with a Tat-specific hyperimmune serum. A distinct immunofluorescence signal was observed in cells infected with WSN/TatΔ_51–59_ virus, whereas no signal was detected with the control WSN virus (Figure 2).

### 3.2. Virulence of WSN/TatΔ_51–59_ Virus

To assess the growth properties of the recombinant WSN/TatΔ_51–59_ virus* in vivo*, we first examined the ability of the virus to replicate in the respiratory tracts of BALB/c mice. Mice were infected i.n. with 10^5^ PFU of WSN/TatΔ_51–59_ virus or with 10^3^ PFU of WSN virus and the viral load was determined in the lungs (Figure 3(b)). Virus was not detected in any mice inoculated with WSN/TatΔ_51–59_ virus at the indicated time points, and the absence of clinical and pathological signs, such as body weight loss or pneumonia, showed that WSN/TatΔ_51–59_ virus was highly attenuated in mice. We also assessed viral replication in mice infected i.vag. with WSN or WSN/TatΔ_51–59_ virus. Viral replication in the vaginal mucosal tissues was evident with the highest titers of WSN virus present in the vaginal washes of progesterone-treated female BALB/c mice on days 3 to 5, whereas replication of WSN/TatΔ_51–59_ virus was not detected at the indicated time points (Figure 3(c)). Taken together, these data show that the recombinant virus was essentially unable to cause a productive infection* in vivo*.

### 3.3. Tat-Specific T Cell Responses

**Figure 4:**
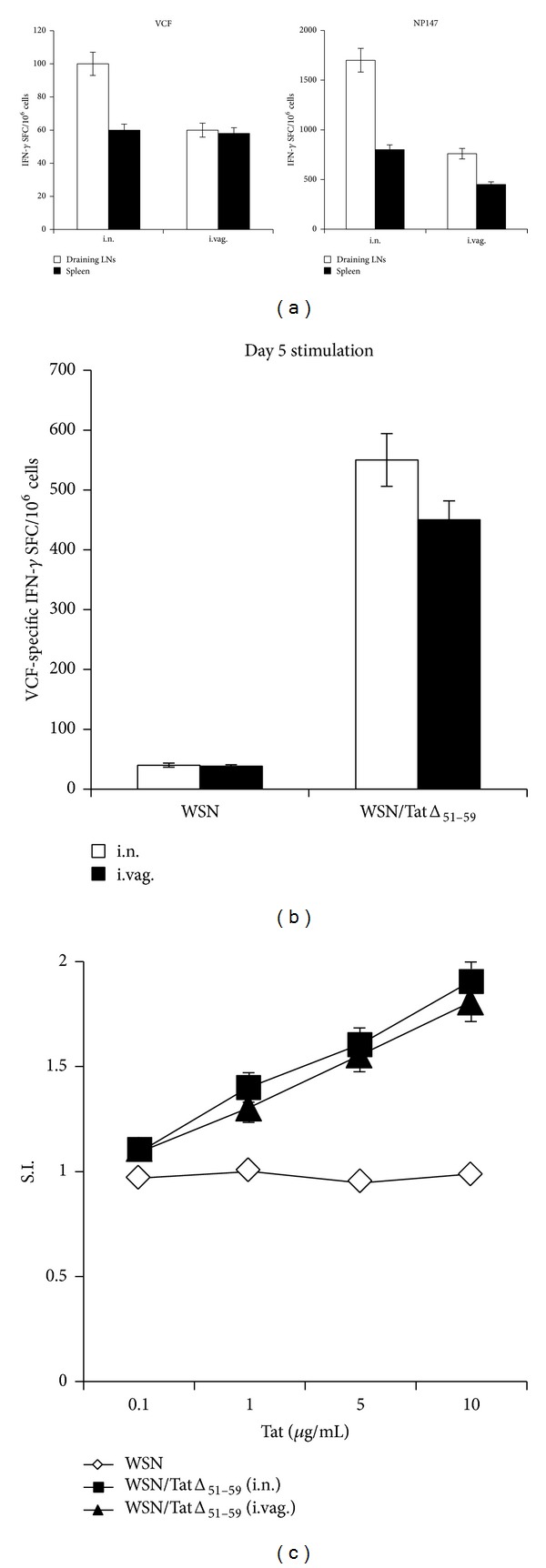
Tat-specific T cell responses in mice vaccinated with WSN/TatΔ_51–59_ virus. BALB/c mice (*n* = 5/group) were immunized i.n. or i.vag. with WSN/TatΔ_51–59_ virus and 7 days later Tat-specific T cell responses were measured in cells from distinct pools of draining lymph nodes, MLN and ILN, respectively, and freshly isolated splenocytes by use of an* ex vivo* IFN-*γ* ELISPOT assay with the indicated peptides (a). Splenocytes from mice immunized with WSN/TatΔ_51–59_ or WSN virus were cultured for 5 days in the presence of VCF and then washed for the IFN-*γ* ELISPOT assay. The values reported are those obtained from stimulated cells minus the background from nonstimulated cells. Bars represent the mean values ± SD of triplicate wells. The data are representative of three independent experiments that gave similar results (b). Splenocytes from the above mice were restimulated in the presence of different concentrations of Tat protein for 5 days and the resulting proliferation was assessed by measuring [^3^H] thymidine incorporation. Results are expressed as the ratio between values (averages of quintuplicates from stimulated and nonstimulated cultures). SI = stimulation index (c).

We next determined whether the engineered TatΔ_51–59_ protein was immunogenic following infection with WSN/TatΔ_51–59_ virus. Our previous studies showed that i.vag. immunization of mice with recombinant influenza viruses bearing HIV-1 antigens induces a vigorous antigen-specific CD8+ T cell immune response that is detectable in both mucosal and systemic compartments [17, 24]. Therefore, we evaluated the T cell responses elicited by the WSN/TatΔ_51–59_ virus in BALB/c mice after single i.n. or i.vag. immunization with WSN/TatΔ_51–59_ or WSN virus, according to the immunization protocols described in the Materials and Methods. Mice were sacrificed 7 days p.i., and the frequencies of Tat-specific IFN-*γ*-producing T cells from regional lymph nodes and spleens were determined by use of an* ex vivo* ELISPOT assay with the VCF Tat peptide, which contains a H-2K^d^-restricted CTL epitope and a CD4+ T cell epitope [22, 23]. VCF-specific IFN-*γ* production was detected in cells derived from both draining lymph nodes and spleens of WSN/TatΔ_51–59_-immunized mice (Figure 4(a)), whereas the draining lymph nodes and spleens derived from the WSN-immunized mice and naive mice did not show any HIV-specific response (data not shown). CD8+ T cell responses specific for the influenza nucleoprotein immunodominant NP147 epitope (H-2K^d^) were also measured to monitor the immune response against the viral vector [21], and higher reactivity, compared with the VCF epitope, was detected. Conversely, there were 2-fold fewer NP147-specific CD8+ T cells in WSN/TatΔ_51–59_-immunized mice, compared with those measured in WSN-immunized mice, thus confirming the low replication capacity of the recombinant virus (data not shown). Under all of the conditions tested, no reactivity was detected when we used unrelated peptides that bind to the same MHC molecules, indicating that a specific immune response was induced in the immunized mice.

The frequencies of Tat-specific IFN-*γ*-producing T cells were also determined after 5 days of stimulation* in vitro* with the VCF peptide. Specific T cell expansion and IFN-*γ* production were observed only in the WSN/TatΔ_51–59_-immunized mice (for both immunization routes), confirming the immunogenicity of the recombinant influenza virus vector (Figure 4(b)). Finally, the cellular immune responses induced by a single immunization with WSN/TatΔ_51–59_ virus were also evaluated by assessing the cell proliferation of spleen cells after* in vitro* stimulation with the Tat protein for 5 days. Antigen-specific and dose-dependent cell proliferation was detected in WSN/TatΔ_51–59_-immunized mice, but not in untreated splenocytes or the lymphocytes of mice immunized with the parental WSN virus (Figure 4(c)).

### 3.4. Induction of Tat-Specific Antibodies by WSN/TatΔ_51–59_ Virus Immunization

**Figure 5:**
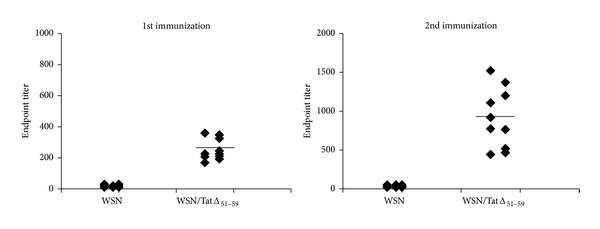
Humoral immune response. Mice (*n* = 10/group) were i.n. infected with WSN or WSN/TatΔ_51–59_ virus. Tat-specific IgG antibodies were detected at 21 days after immunization and at 14 days after boost by use of an ELISA. Results represent the endpoint values for individual mice.

The ability to induce a humoral immune response was determined in BALB/c mice after single dose and double doses of i.n. immunization with WSN/TatΔ_51–59_ or with WSN virus as a control. Mice were bled and sera analyzed for the presence of specific anti-Tat IgG antibodies by use of an ELISA. All mice immunized with a single dose of WSN/TatΔ_51–59_ virus developed a detectable anti-Tat antibody response (Figure 5). A booster dose increased the antibody titers (right panel) in these mice, whereas no anti-Tat-specific response was detected in mice immunized with WSN virus. Tat delivery by the recombinant WSN/TatΔ_51–59_ virus thus induced anti-Tat humoral responses even after a single immunization.

## 4. Discussion 

Vaccines capable of inducing long-term mucosal and systemic immune responses would protect against HIV-1. Accordingly, various delivery systems and vaccination regimens have been tested in preclinical and clinical studies. Here, we successfully generated a recombinant influenza virus bearing a TatΔ_51–59_ protein at the N-terminus of the HA protein that was transported via the classical secretory pathway, exposed at the cell surface, and incorporated into virions. The recombinant virus was highly attenuated in mice, and a single dose was able to induce Tat-specific humoral and cellular immune responses. These findings provide evidence for the immunogenicity of the chimeric HA/Tat protein that could form the basis for a new vaccine formulation against HIV-1.

Among HIV-1 proteins, Tat is a promising antigen for the formulation of a therapeutic or prophylactic vaccine against AIDS [25, 26]. In preclinical studies with macaques, immunization with Tat-based vaccines (both native protein and plasmid DNA) was shown to be immunogenic and effective in controlling virus replication and blocking disease onset [27–30]. Moreover, in monkeys, administration of a trimeric Env protein in the presence of a Tat subunit vaccine prevented virus spread from the portal of entry to the regional lymph nodes [31]. In humans, a Tat-based vaccine has demonstrated promising efficacy in a phase II therapeutic trial in HAART-treated subjects [13]. Thus, the development of innovative approaches of immunization designed to elicit systemic and mucosal immunity specific to Tat may improve its immunogenicity and contribute to the establishment of innovative Tat-based vaccines combined with other HIV antigens.

Recombinant influenza viruses have been used as experimental vaccine vectors to express a variety of antigens and are known to be immunogenic in mice [14, 15]. In particular, relatively large antigens can be inserted into the HA protein and functionally expressed during infection in mice [32]. Moreover, in a recent study we showed that mucosal immunization of mice with WSN/CKG virus carrying an HIV polyepitope that contained the PCLUS3-P18 peptide in its HA induced protective immunity against challenge with recombinant vaccinia virus vPE16 expressing gp160 HIV-1 IIIB [24]. In this study, we generated WSN/TatΔ_51–59_ virus bearing the chimeric HA/Tat protein with a 9-amino acid deletion in the basic region of Tat, which contains motifs recognized by furin-like endoproteases. This deletion prevents the rare but possible intragenic recombination events involving the multibasic residues at the cleavage site of the chimeric HA-Tat and thus the emergence of a highly pathogenic influenza virus [33]. Moreover, there is evidence that the conserved basic domain of Tat plays an important role in interactions between both viral and host cellular factors, thus mediating many multifunctional properties, such as binding to heparan sulfate proteoglycans, protein transfer across the cell membrane, nucleolar localization, and transactivating transcriptional activities [34]. By removing this basic region, we tried to prevent potential effects of the chimeric fusion protein on membrane trafficking and thus on the assembly and release of the recombinant virus.

Although we were able to generate the WSN/TatΔ_51–59_ recombinant virus, the presence of the Tat protein affected the growth properties of the virus in tissue culture and to a greater extent* in vivo*. This is probably due to the Tat protein itself because the insertion of unrelated larger antigens in HA did not affect the viral replication* in vitro,* as described elsewhere [32]. Indeed, the relatively high numbers of CD8+ T cells specific for the influenza nucleoprotein immunodominant NP147 epitope measured in the vaccinated mice suggest that the WSN/TatΔ_51–59_ virus was able to replicate to some extent* in vivo.* Although no productive virus infection was detected with the inoculum dose used, WSN/Tat virus was nevertheless able to elicit Tat-specific antibodies after a single mucosal immunization of BALB/c mice. Overall, these results support the evidence that the HA/TatΔ_51–59_ chimeric protein was transported via the secretory pathway, exposed at the cell surface, and incorporated into virions. It is also important to point out that WSN/TatΔ_51–59_ virus successfully induced a Tat-specific T cell response, given that Tat-specific IFN-*γ* production was detectable* ex vivo* on fresh splenocytes of singly immunized mice. Moreover, substantial Tat-specific IFN-*γ* production was also detected upon single i.vag. immunization with WSN/TatΔ_51–59_ virus, thus confirming previous data on the efficacy of this route for recombinant influenza viruses [17, 24].

The ease of production of influenza virus-based immunogens and the availability of different vaccine formulations make the recombinant influenza viruses an effective vaccine platform for the delivery of foreign antigens and thus potentially suitable for heterologous prime-boost regimens. Inactivated influenza virus vaccines are excellent inducers of virus-specific serum antibodies, and the insertion of foreign antigens into the influenza HA chimeric proteins could provide potential vaccine candidates to induce HIV-specific immunity in target individuals. Additionally, one of the most interesting and feasible application would be the use of recombinant influenza virus-derived virosomes. Surface display of foreign antigens, for example, the Tat protein, in the virosomal formulations would allow the generation of Tat-specific antibodies as well as the induction of cellular immunity. In this case, even the bias of the preexisting immunity to the influenza virus in the human population would favor the Tat-specific immune responses, because memory CD4+ T cells and antibodies against influenza glycoproteins enhance the capacity of virosomes to act as carriers for the delivery of antigens for cellular immune responses [35–38]. Synthetic peptides representing T cell epitopes can also be packaged during the reconstitution process into recombinant influenza virus-derived virosomes engineered with chimeric HAs fused to different HIV antigens, thus providing a modular and flexible antigen delivery platform suitable for prophylactic and therapeutic vaccines. It should be noted, however, that influenza virus infection requires binding to cell surface receptors, endocytosis, and a low pH-dependent fusion process. Thus, the design of recombinant HA proteins should always take into account the steric hindrance and conformational alterations that could interfere with the virus infection process and transport of the chimeric HA to the cell surface and consequently with the rescue of a genetically stable recombinant influenza virus.

In conclusion, our results provide a proof-of-concept for a systematic approach based on recombinant influenza viruses for promising immunogens to be further investigated as potential vaccine candidates for HIV-1.
